# Identification of a novel mutation in pseudohypoparathyroidism type Ia in a Chinese family: A case report

**DOI:** 10.1097/MD.0000000000019965

**Published:** 2020-05-22

**Authors:** Yuchen Tang, Fenping Zheng, Xihua Lin, Qianqian Pan, Lin Li, Hong Li

**Affiliations:** Department of Endocrinology, The Affiliated Sir Run Run Shaw Hospital, School of Medicine, Zhejiang University, Hangzhou, Zhejiang Province, P.R. China.

**Keywords:** Albright hereditary osteodystrophy, GNAS, parathyroid hormone resistance, pseudohypoparathyroidism type Ia

## Abstract

**Introduction:**

Pseudohypoparathyroidism (PHP) indicates a group of rare disorders characterized by end-organ resistance to various hormones, primarily parathyroid hormone (PTH). One of its most common type is PHP-Ia, which is caused by maternally inherited inactivating mutations in *GNAS.* In this report, we present a Chinese girl with typical features of PHP-Ia and a novel mutation of the *GNAS* gene.

**Patient concerns:**

A 9-year-old Chinese girl presented with recurrent epileptic seizure.

**Diagnosis:**

Biochemical and imaging findings were consistent with PHP-Ia, including typical Albright hereditary osteodystrophy phenotype (short stature, round face, brachydactyly, and mild mental retardation), PTH resistance (hypocalcemia, hyperphosphatemia, elevated serum PTH, and multiple intracranial calcification) and thyroid stimulating hormone resistance (elevated serum thyroid stimulating hormone).

**Interventions:**

The patient was given 1α-hydroxylated vitamin D (calcitriol, 0.5 ug/d), calcium carbonate and vitamin D_3_ tablets (1.5 g/d, including 600 mg calcium and 125 IU vitamin D_3_). DNA analysis of the *GNAS* gene was performed for the whole family.

**Outcomes:**

Investigation of the *GNAS* gene revealed a novel mutation c.313delG (p.Glu105Lysfs∗7) in the patient, as well as her mother. So the diagnosis of PHP-Ia was confirmed.

**Conclusion:**

The study further expands the spectrum of known *GNAS* mutations associated with PHP and lay emphasis on the genetic analysis of *GNAS* gene for identifying genetic abnormalities as well as making diagnosis and differentiation of various subtypes of PHP.

## 1 Introduction

Pseudohypoparathyroidism (PHP) indicates a group of rare disorders characterized by end-organ resistance to parathyroid hormone (PTH) leading to hypocalcemia, hyperphosphatemia, and elevated serum PTH.^[1]^ According to urinary cAMP and phosphaturic response to exogenous PTH administration, PHP is divided into type I and type II.^[2]^ The former shows a blunted urinary cAMP and phosphaturic response while the latter has normal urinary cAMP excretion but blunted phosphaturic response.^[3]^ Moreover, PHP-I is further classified into 3 different subtypes based on hormone resistance patterns and the presence or absence of Albright hereditary osteodystrophy (AHO) phenotype,^[4]^ which includes short stature, obesity, round facies, brachydactyly, subcutaneous calcification, and mental retardation.^[5]^ Patients with PHP-Ia have AHO phenotype and multi-hormone resistance in addition to PTH, including thyroid stimulating hormone (TSH), gonadotropins, and growth hormone releasing hormone.^[1]^ PHP-Ib is classically characterized by PTH resistance but without the features of AHO.^[6]^ Patients with PHP-Ic display similar clinical manifestations to PHP-Ia but normal activity of the alpha-subunit of the stimulatory G protein (Gsα).^[7]^ Patients with the features of AHO but without hormone resistance are referred to as pseudopseudohypoparathyroidism (PPHP).^[5]^

Indeed, PHP-Ia and PPHP are caused by inactivating mutations within the *GNAS* gene, which is located on the long arm of chromosome 20 in humans and contains 13 exons.^[8]^ All exons can be affected by loss-of-function alterations, of which small insertions/deletions and amino acid substitutions are most commonly found.^[9]^ The mutation leads to a dramatic reduction in Gsα expression or activity in certain tissues, thus resulting in abnormal signaling of cAMP-dependent pathways.^[10]^ The mutation is maternally inherited in PHP-Ia while paternally inherited in PPHP.^[11]^

We herein report a 9-year-old girl with PHP-Ia resulted from a novel mutation c.313delG in the *GNAS* gene. Further investigation of the family revealed the same mutation in the patient's mother.

## 2 Case presentation

A 9-year-old girl was admitted to the Sir Run Run Shaw Hospital with recurrent epileptic seizure. The symptoms first appeared 3 years ago, including vigorous limb spasm, foaming at the mouth, locked jaw, rolled eyes, and loss of consciousness. It lasted about 20 minutes and relieved automatically without incontinence or prodromal symptoms. Similar situation recurred for a total of 5 times and she was diagnosed with PHP by the local hospital due to hypocalcemia, hyperphosphatemia, elevated serum PTH, and multiple intracranial calcification. Physical examination showed short stature (height 119 cm, −2SD∼−3SD), round face, brachydactyly with short metacarpals, metacarpal sign (+) and mild mental retardation. Her weight was 26.5 kg (−1SD∼M), thus not meeting the criteria for obesity. Family history revealed that her parents and her younger brother stayed normal except her mother had short stature, which might suggest AHO.

**Figure 1:**
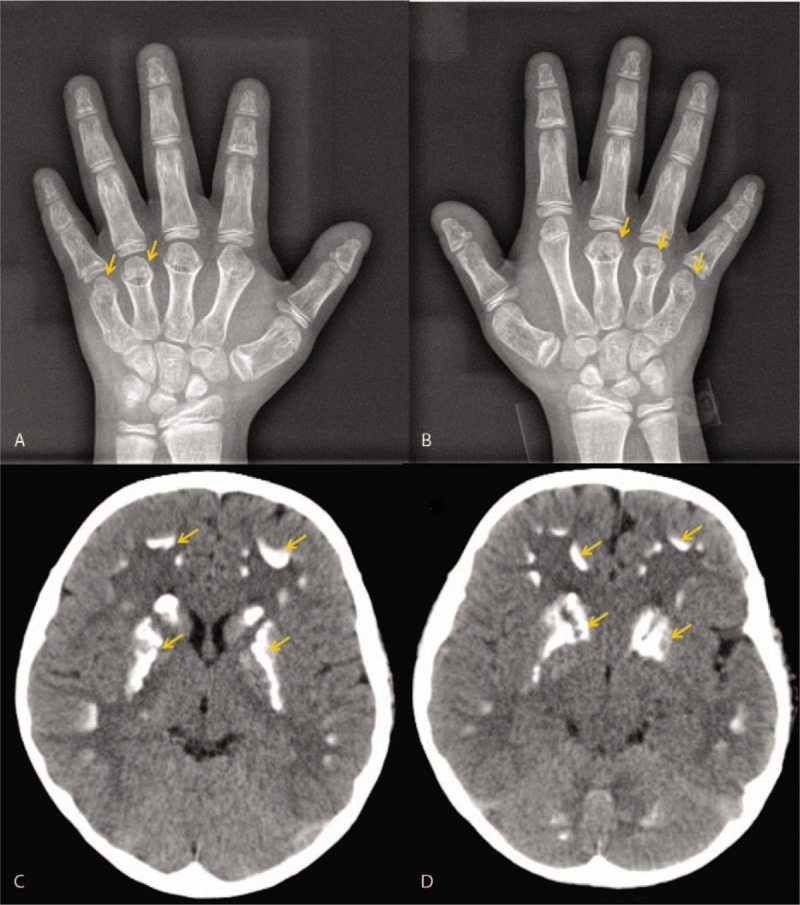
Radiograph of the hands (A: left hand, B: right hand) showing shortened metacarpals (arrow). Cranial computed tomography scan (C, D) demonstrated bilateral calcifications in various regions of cerebrum and cerebellum, especially the basal ganglia (arrow).

Laboratory tests revealed hypocalcemia (1.45 mmol/L [2.20–2.70]), hyperphosphatemia (2.74 mmol/L [0.8–1.6]), elevated serum PTH (671.9 ng/L [15.0–65.0]), decreased 24-hour urinary calcium and phosphorous (0.141 mmol and 0.846 mmol respectively, [2.5–7.5] and [2.10–8.19], respectively). She also showed lightly elevated plasma TSH (6.24 mIU/L [0.35–4.94]) and normal thyroid hormone levels (TT3 1.04 ng/mL [0.57–1.59]; TT4 5.36 μg/dL [4.87–11.72]; FT3 2.95 pg/mL [1.71–3.71]; FT4 1.06 ng/dL [0.70–1.48]), as well as moderately elevated thyroperoxidase antibody level of 251.35 IU/mL ([0.00–5.61]). Follicle stimulating hormone, luteinizing hormone, growth hormone and IGF-1 levels were normal. Hands’ X-ray demonstrated short 4th and 5th metacarpals on the left and 3th, 4th, and 5th on the right (Fig. 1A and B). Cranial computed tomography scan demonstrated bilateral calcifications in various regions of cerebrum and cerebellum, especially the basal ganglia (Fig. 1C and D).

Since the patient showed a typical AHO phenotype and typical laboratory and radiological findings, although the PTH infusion testing was impeded by the lack of commercially available PTH and Gsα protein activity was not measured, the diagnosis of PHP-Ia was primarily considered. She was then given 1α-hydroxylated vitamin D (calcitriol, 0.5 ug/d) and calcium carbonate and vitamin D_3_ tablets (1.5 g/d, including 600 mg calcium and 125 IU vitamin D_3_).

According to the studies up to now, PHP-Ia is caused by maternally inherited inactivating mutations in the 13 exons of the *GNAS* gene.^[9]^ To further support the diagnosis of PHP-Ia and to make differential diagnosis from other subtypes of PHP, we performed DNA analysis of the *GNAS* gene. After obtaining informed consent from both parents, genomic DNA was extracted from peripheral blood samples of the patient and her parents using the RelaxGene Blood DNA System following the manufacturer's instructions (Tiangen, SanJose, CA). All 13 coding exons and their flanking introns of the *GNAS* gene were amplified using primer sets according to literature).^[12]^ Polymerase chain reaction was performed with a thermal cycler (Applied Biosystems, Foster City, CA) and the protocol is available on request. Polymerase chain reaction fragments amplified from genomic DNA were analyzed by electrophoresis using 1% agarose gel containing gelred and the result was visualized using UV light. Direct sequencing was performed by Tsingke biological technology. Mutations were compared in the Human Gene Mutation Database (http://www.hgmd.cf.ac.uk/ac/all.php) and the Leiden Open Variation Database (www.lovd.nl/GNAS).

**Figure 2:**
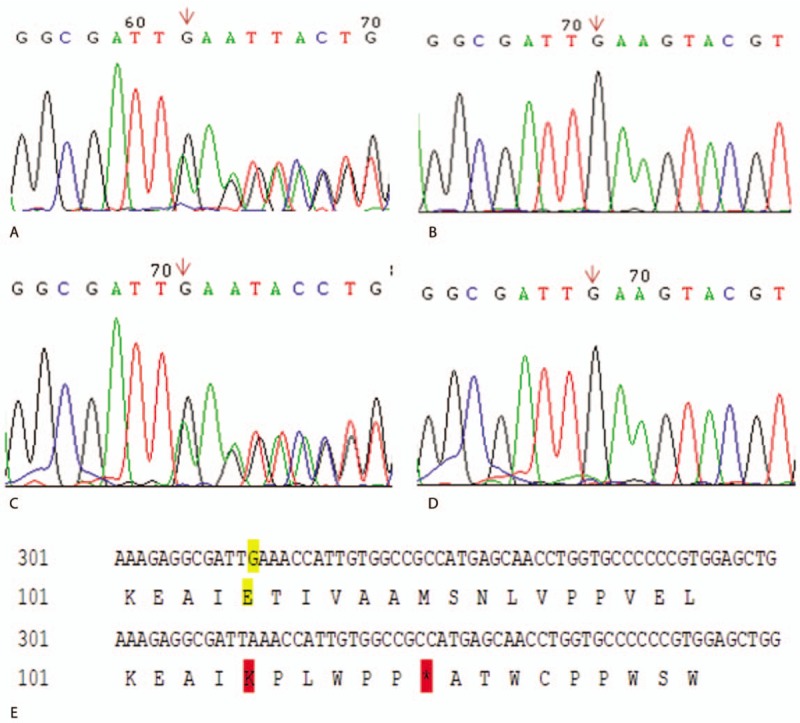
Sequencing analysis of exon 4 shows 1-base pair deletion in exon 4 (arrow) in the proband (A) and her mother (C). The mutation was absent in her father (B) and her brother (D). Normal nucleotide and amino acid sequence (E, above) and aberrant ones (E, below).

A novel heterozygous 1-base pair (bp) deletion in exon 4 (c.313delG) was found in the proband and her mother (Fig. 2). The deletion of this nucleotide sequence is predicted to cause a frameshift mutation and might lead to a premature stop codon in the new frame (Fig. 2E), thus producing truncated gene products (p.Glu105Lysfs∗7). The mutation was absent in the unaffected family members, including the patient's father and her brother, thereby indicating that the mutation is on the maternal allele. However, her grandparents’ blood samples were not available, so whether the mother has a de novo mutation involving the paternal allele or that she inherited the mutation from the grandfather is unknown.

## 3 Discussion

In this report, we present a Chinese girl with typical features of AHO and resistance to PTH. Direct sequencing of 13 exons of *GNAS* gene in the family members demonstrated a novel heterozygous mutation in *GNAS* in the patient and her mother but not her father. Thus the diagnosis of PHP-Ia was confirmed in the patient. However, the patient's mother only had short stature but no hormone resistance, which suggested PPHP.

The elevated serum TSH indicated possible existence of TSH resistance. However, Hashimoto's thyroiditis cannot be excluded due to moderately elevated thyroperoxidase antibody level. The elevated serum TSH can also suggest subclinical hypothyroidism, which can be a manifestation of Hashimoto's thyroiditis. Zeng et al^[13]^ have reported a case of an individual with PHP, Turner syndrome and Hashimoto thyroiditis. A fine-needle aspiration might help to distinguish between TSH resistance and Hashimoto thyroiditis.

The mechanism underlying PHP-Ia and PPHP is maternal or paternal inactivating mutations in *GNAS* respectively, which encodes Gsα, thus leading to abnormal signaling of cAMP-dependent pathways.^[11]^ As in our case, a novel heterozygous 1- bp deletion in exon 4 (c.313delG) was found in the proband and her mother which is predicted to cause a frameshift mutation and might lead to a premature stop codon in the new frame, thus producing truncated gene products (p.Glu105Lysfs∗7). The different clinical presentation in the daughter (PHP-1a) and the mother (PPHP), who harbors the same mutation, can be explained by the parental genomic imprinting which leads to a difference in hormone responsiveness in certain tissues, such as the renal proximal tubule and the thyroid. In other tissues, no imprinting would be expected and a 50% reduction of Gsα activity is still sufficient for maintaining normal signaling activity in most cells but leads to haploinsufficiency in others tissues involved in the AHO phenotype, such as the growth plate.^[14]^ Many PHP-1a and PPHP patients have a similar heterozygous loss-of-function mutation in the *GNAS* gene; however, the severity of AHO is variable. The variability of a phenotype could be due to epigenetic alterations, altered transcriptional regulation, or effects of other genes.^[15]^

About 70% patients with PHP-Ia were identified with mutations in the 13 exons of the *GNAS* gene.^[9]^ In order to support the diagnosis of PHP-Ia and to make differential diagnosis from other subtypes of PHP, genetic analysis is necessary. In this study, a novel heterozygous 1 bp deletion in exon 4 (c.313delG) was identified, which might be useful for screening other family members in order to avoid late or misdiagnosis. In addition, the detection of the mutation can be used for prenatal diagnosis of the patient's offspring.

There are over 200 reported mutations scattered across the 13 exons that encode Gsα, including small insertions/deletions, amino acid substitutions, nonsense mutations, and point mutations, most of which can be found in the Human Gene Mutation Database and the Leiden Open Variation Database.^[16]^ Small insertions/deletions causing frameshift mutation makes up nearly half of all the mutations, and a 4-bp deletion within exon 7 is considered a hot spot.^[17]^ There are previous reports of Chinese patients with PHP-Ia and PPHP who were confirmed by genetic analysis. Although many mutations have been identified in *GNAS*, to our knowledge, c.313delG (p.Glu105Lysfs∗7) is novel.

More studies are needed in order to further assess whether this unique mutation resulted in the dysfunction of Gsα. In the next step, we plan to introduce the mutation into a rat *GNAS* plasmid and perform functional studies to assess the level of cAMP activity associated with this mutation.

In conclusion, our study possibly further expands the spectrum of known *GNAS* mutations associated with PHP and lay emphasis on the genetic analysis of *GNAS* gene for identifying genetic abnormalities as well as making diagnosis and differentiation of various subtypes of PHP.

## Acknowledgment

The authors thank the family for participating and supporting this study.

## Author contributions

**Data curation:** Yuchen Tang, Fenping Zheng, Lin Li, Hong Li.

**Formal analysis:** Yuchen Tang, Fenping Zheng, Lin Li, Hong Li.

**Investigation:** Yuchen Tang, Xihua Lin, Qianqian Pan.

**Methodology:** Yuchen Tang, Xihua Lin, Qianqian Pan.

**Writing – original draft:** Yuchen Tang.

**Writing – review and editing:** Fenping Zheng, Xihua Lin, Hong Li.
